# Safety and Efficacy of Bromodomain and Extra-Terminal Inhibitors for the Treatment of Hematological Malignancies and Solid Tumors: A Systematic Study of Clinical Trials

**DOI:** 10.3389/fphar.2020.621093

**Published:** 2021-01-26

**Authors:** Yanli Sun, Jie Han, Zhanzhao Wang, Xuening Li, Yanhua Sun, Zhenbo Hu

**Affiliations:** ^1^ Laboratory for Stem Cell and Regenerative Medicine, Affiliated Hospital of Weifang Medical University, Weifang, China; ^2^ Department of Laboratory Medicine, Weifang Medical University, Weifang, China; ^3^ Weifang Medical University, Weifang, China; ^4^ Department of Laboratory Medicine, Weifang People’s Hospital, Weifang, China; ^5^ Department of Hematology, Weifang People’s Hospital, Weifang, China; ^6^ Department of Hematology, Affiliated Hospital of Weifang Medical University, Weifang, China

**Keywords:** bromodomain and extra-terminal inhibitor, hematological malignancy, solid tumor, safety, efficacy

## Abstract

**Background:** The upregulated expression of BET proteins is closely associated with the occurrence and development of hematological malignancies and solid tumors. Several BET inhibitors have been developed, and some have been in phase I/II of clinical trials. Here, the safety, efficacy, and pharmacodynamics of ten BET inhibitors currently in clinical trials were evaluated.

**Methods:** We retrieved and reviewed published reports on the clinical trials of twelve BET inhibitors including AZD5153, ABBV-075, BMS-986158, CPI-0610, GSK525762, OTX-015, PLX51107, INCB054329, INCB057643, FT-1101, CC-90010, and ODM-207 for patients with hematological malignancies and solid tumors and summarized their published target genes.

**Results:** In the monotherapy of BET inhibitors, the most common and severe (grade ≥3) hematological adverse events (AEs) are thrombocytopenia, anemia, and neutropenia. The most common non-hematological syndromes are diarrhea, nausea, fatigue, dysgeusia, and decreased appetite, while the most severe AE is pneumonia. Additionally, *T*_max_ of these BET inhibitors was between 0.5–6 h, but the range for *T*_1/2_ varied significantly. According to published data, the rates of SD, PD, CR and PR were 27.4%, 37.6%, 3.5%, and 5.7%, respectively, which is not very satisfactory. In addition to BRD4, oncogene MYC is another common target gene of these BET inhibitors. Ninety-seven signaling pathways may be regulated by BET inhibitors.

**Conclusion:** All BET inhibitors reviewed in our study exhibited exposure-dependent thrombocytopenia, which may limit their clinical application. Moreover, further efforts are necessary to explore the optimal dosing schemes and combinations to maximize the efficacy of BET inhibitors.

## Introduction

Acetylation of lysine residues is an important post-translational modification for DNA binding proteins, especially histone (Verdin and Ott, 2015). Acetylation of histone leads to the decompaction of chromatin, which allows transcription factors, DNA polymerases, or DNA repair components to get access to DNA (Bechter and Schöffski, 2020).

Histone acetylation is recognized by Bromodomain and extra-terminal (BET) proteins, a subclass of Bromodomain (BRD) containing proteins. The BET proteins consist of four members: BRD containing BRD2, BRD3, BRD4, and BRDT (Lovén et al., 2013). They all harbor two bromodomain binding domains BD1 and BD2 at their N-terminal site as well as an extra terminal (ET) domain, while BRD4 and BRDT contain an additional C-terminal domain (CTD) (Hsu and Blobel, 2017). Among these domains, BD1 and BD2 contain an amino acidic hydrophobic pocket, which is the binding site of the acetylated lysine residues on histone (Alqahtani et al., 2019). The ET domain is necessary for the recruitment of the components of the transcriptional complex, while the CTD is responsible for the recruitment of positive elongation factor (P-TEFb).

Except for BRDT, the other BET proteins BRD2, BRD3, and BRD4 are expressed ubiquitously in mammalian cells and play a vital role in transcriptional regulation. BRD2 and BRD3 mainly participate in the regulation of the cell cycle through the recruitment of E2F transcription factor and facilitating the transcription of RNA polymerase II gene (Crowley et al., 2004; Peng et al., 2007; LeRoy et al., 2008; Stonestrom et al., 2015). Unlike BRD2 and BRD3, aside from maintaining chromatin acetylation status, BRD4 can also regulate the transcription of growth-promoting genes through recruiting PTEFb (Houzelstein et al., 2002) or NSD3 (Rahman et al., 2011).

Altered histone acetylation results in the aberrant transcription of cancer-related genes. It has been proven that BET proteins enhance the expression of the oncogene MYC, which participates in the tumorigenesis of multiple malignancies including acute myeloid leukemia (Li et al., 2020), acute lymphoblastic leukemia, and lymphoma (De Barrios et al., 2020). Moreover, BET proteins play a critical role in oncogenic rearrangements comprising oncogenic fusion proteins in several types of cancer (Muller et al., 2011). Thus, we can infer the therapy of targeting BET proteins may be effective in certain types of cancers.

Since the discovery of the first two BET inhibitors GSK525762 and JQ1 (Filippakopoulos et al., 2010; Nicodeme et al., 2010), a variety of new BET inhibitors have been developed. They serve a multitude of functions in various tumors, diabetes, chronic kidney failure, coronary artery disease, and other infectious diseases (Gilan et al., 2020; Morse et al., 2018; Cochran et al., 2019). Their binding site is the BRD-acetyl binding pocket, hence blocking the binding of BET protein to the enhancers and promoters, mainly super-enhancers. Exposure of tumor cells to BET inhibitors results in decreased expression of BRD4 and downregulates transcription of key oncogenes, such as *MYC*, *FOSL1*, and *CDK6* (Donati et al., 2018). It is important to note they have different affinity and selectivity for either BD1 or BD2 (Alqahtani et al., 2019). A diverse range of side effects from BET inhibitors has been reported in different studies. In this study, we analyzed current clinical trials and summarized the safety, efficacy, pharmacokinetics, and target genes of BET inhibitors including AZD5153, ABBV-075, BMS-986158, CPI-0610, GSK525762, OTX-015, PLX51107, INCB054329, INCB057643 (Supplementary Figure S1), ODM-207 (Lindqvist et al., 2019), CC-90010, and FT-1101 (their chemical structure is currently unavailable).

## Methods

### Study Design, Search Strategy and Study Selection

Our study followed the guidance of the preferred reporting items for systematic reviews and meta-analysis (PRISMA) statements (Stewart et al., 2015). The questions mentioned here were organized according to the population, intervention, comparison, and outcome (PICO) format rules—1) population: adult patients with malignant tumor; 2) intervention: treated with one of BET inhibitors; 3) comparison: with/without control; 4) outcomes: adverse events (AEs) and efficacy (including CR, PR, SD, and PD) after using drugs and pharmacodynamics including the steady-state time to reach the maximum plasma concentration (*T*_max_) and the terminal elimination half-life (*T*_1/2_). A literature search was also performed in the databases of PubMed, Embase, and Cochrane Library (before December 10, 2020). The following keywords and derived combinations were used without any automatic filters: BET inhibitor or BRD inhibitor.

### Quality Assessment

Two independent researchers (HJ and SYH) used the methodological index for non-randomized studies (MINORS) (Slim et al., 2003) to assess methodological quality and standard of outcomes reported in the included studies. These items are scored 0, 1, or 2. Score 0 represents no data was found in the study; score 1 indicates not adequate data reported; Score 2 reflects adequate data reported. The full score 16 indicated the study was of high quality.

### Inclusion and Exclusion Criteria

The eligibility criteria in the study were as follows: 1) clinical trial; 2) the adult patients with malignant tumors enrolled in these trials were confirmed by the corresponding diagnostic criteria; 3) they were treated with BET inhibitors, regardless of any treatment before; 4) full data of the safety and/or efficacy was available in the articles.

The exclusion criteria were as follows: 1) cell experiment or animal experiment; 2) articles without raw data; 3) articles sharing identical raw data.

### Data Extraction

Extracted data were as follows: 1) the fundamental information including the type of BET inhibitor, first author, registered number and phase of clinical trials, publication date, number of participating patients, cancer type and age; 2) the characteristics of adverse events (AEs) of all grades and grade ≥3; 3) survival indicators including SD (stable disease), PD (progressive disease), CR (complete remission), and PR (partial response); 4) pharmacodynamics including *T*_max_ and *T*_1/2_. Some information was not included in this study due to limited data.

Additionally, the target genes of these BET inhibitors from published articles were summarized, and the related Kyoto Encyclopedia of Genes and Genomes (KEGG) pathway and Gene Ontology (GO) enrichment analysis were performed using online software Metascape (https://metascape.org/gp/index.html) (Zhou et al., 2019).

### Statistical Analysis

The data of survival and AEs were analyzed by the Comprehensive Meta-Analysis program 2 (Biostat, Englewood, NJ) in this study. The event rate and 95% confidence interval (CI) of major AEs including all grades and grade≥3 were evaluated using a statistical threshold of *p* < 0.05. If *I* ^*2*^ ≥ 50% and *p* < 0.05, the random-effect model was chosen in the analysis, otherwise the fixed-effect model was applied. The correlation between the common adverse effects and efficacy was assessed using Spearman correlation analysis.

## Results

### Literature Search

**FIGURE 1:**
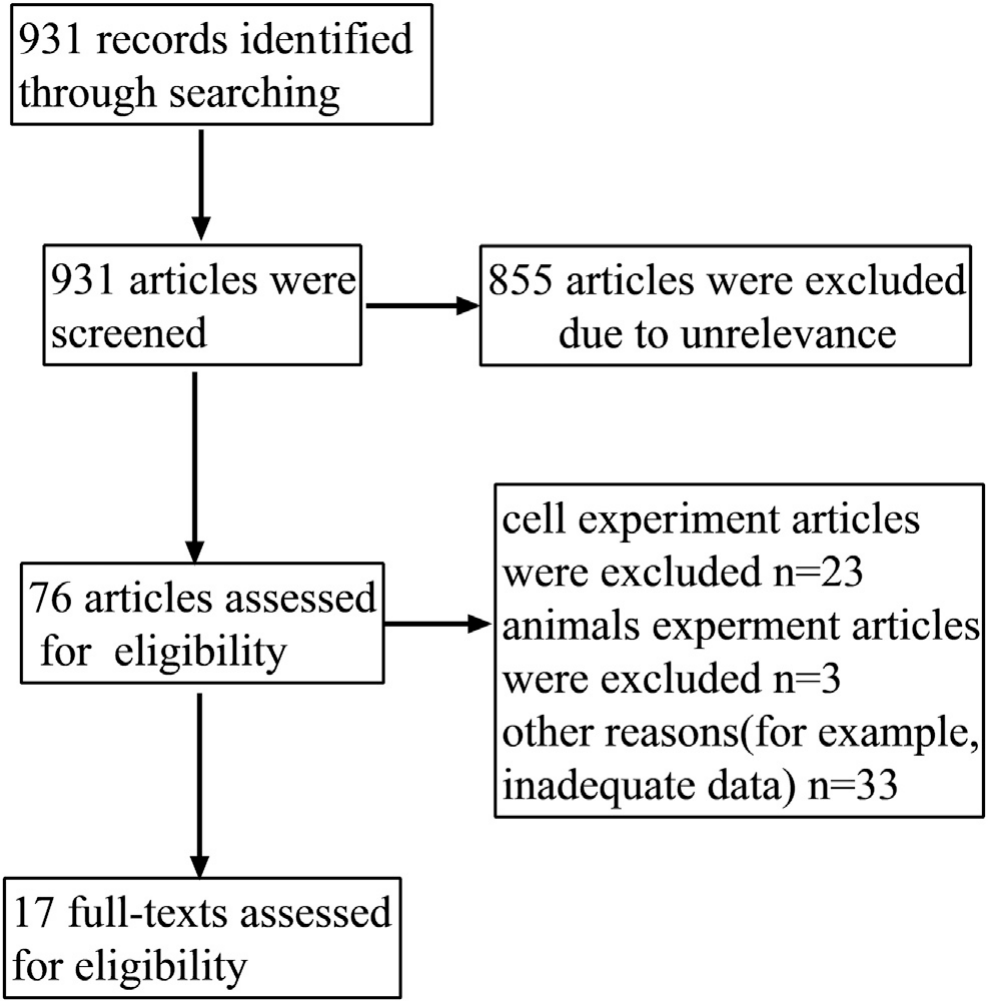
Flow chart of the literature search and selection process.

A total of 931 potentially relevant articles were obtained from the search via PubMed, Embase, and the Cochrane Library before Dec 10, 2020. From the preliminary reading of the titles and abstracts, 855 articles were excluded due to irrelevance. After a careful evaluation of the remaining articles, an additional 76 were rejected due to non-clinical trials or other reasons. Finally, 17 articles were selected for this study. The screening details are shown in Figure 1 and the basic information of the included articles are listed in Table 1.

**TABLE 1:** Basic information of the selected articles.

BET inhibitor (target)	Author	Clinical trial registration number	Phase	Publication year	Number of patients	Study design	Cancer type	Median age (range)
AZD5153 (BRD4)	Wang et al. (2019)	NCT03205176	1	2019	28	Single-arm	R/R malignant solid tumor and lymphoma (*n* = 28)	66.5 (no mention)
ABBV-075 (BRD4)	Piha-Paul et al. (2019)	NCT02391480	1	2019	84	Single-arm	R/R solid tumors (*n* = 72), prostate cancer (*n* = 12)	62.5 (23–83)
BMS-986158 (BRD4)	Hilton et al. (2018)	NCT02419417	1/2a	2018	68	Single-arm	Advanced cancer (*n* = 68)	59 (no mention)
CPI-0610 (BRD4)	Blum et al. (2018)	NCT01949883	1	2018	64	Single-arm	R/R lymphomas (*n* = 64)	No mention
CPI-0610 (BRD4)	Kremyanskaya et al. (2018)	NCT02158858	2	2019	44	Single-arm	Myelofibrosis (*n* = 44)	69 (41–83)
GSK525762 (BRD2/BRD3/BRD4)	Piha-Paul et al. (2020)	NCT01597703	1	2019	65	Single-arm	CRC (*n* = 34), NUT carcinoma (*n* = 29)	≥16
GSK525762 (BRD2/BRD3/BRD4)	Dawson et al. (2017)	No mention	1/2	2017	46	Single-arm	R/R AML (*n* = 41), AML after MDS (*n* = 4), new AML (*n* = 1)	66.5 (24–84)
OTX-015 (BRD2/BRD3/BRD4)	Dombret et al. (2014)	No mention	1	2017	36	Single-arm	AML (*n* = 33), ALL (*n* = 2), RA with a large number of blasts (*n* = 1)	70 (19–85)
OTX-015 (BRD2/BRD3/BRD4)	Berthon et al. (2016)	NCT01713582	1	2016	41	Single-arm	Acute leukemia (*n* = 40), MDS (*n* = 1)	70 (60–75)
OTX-015 (BRD2/BRD3/BRD4)	Amorim et al. (2016)	NCT01713582	1	2016	45	Single-arm	Lymphoma (*n* = 33), myeloma (*n* = 12)	66 (55–72)
OTX-015 (BRD2/BRD3/BRD4)	Massard et al. (2016)	No mention	1b	2016	46	Single-arm	CRPC (*n* = 26), NMC (*n* = 10), KRASmut NSCLC (*n* = 9), ALK^+^ NSCLC (*n* = 1)	No mention
OTX-015 (BRD2/BRD3/BRD4)	Lewin et al. (2018)	NCT02259114	1b	2018	46	Single-arm	NMC (*n* = 10), CRPC (*n* = 26), NSCLC (*n* = 10)	60 (20–79)
PLX51107 (BRD4)	Patnaik et al. (2018)	NCT02683395	2	2018	36	Single-arm	Uveal melanoma (*n* = 11), sarcoma (*n* = 6), NSCLC (*n* = 2), breast cancer (*n* = 2) and CRPC (*n* = 2),other solid tumors (*n* = 13)	60.5 (no mention)
INCB054329 (BRD4)	Falchook et al. (2020)	NCT02431260	1/2	2020	69	Single-arm	Prostate cancer(*n* = 9), CRC (*n* = 8), breast cancer (*n* = 6), ovarian cancer (*n* = 5), lymphoma (*n* = 4), AML (*n* = 3), pancreatic cancer (*n* = 2), MDS (*n* = 1), other (*n* = 31)	63 (18–87)
INCB057643 (BRD4)	Falchook et al. (2020)	NCT02711137	1/2	2020	134	Single-arm	Prostate cancer (*n* = 15), colorectal cancer (*n* = 9), breast cancer (*n* = 18), ovarian cancer (*n* = 16), lymphoma (*n* = 20), AML (*n* = 12), pancreatic cancer (*n* = 7), glioblastoma (*n* = 7), MDS (*n* = 5), other (*n* = 25)	66 (19–84)
FT-1101 (BRD4)	Patel et al. (2019)	NCT02543879	1	2019	94	Single-arm	AML/MDS (*n* = 84), NHL (*n* = 10)	AML/MDS: 74 (35–92), NHL: 7341–82)
CC-90010 (BRD2/4)	Moreno et al. (2020)	NCT03220347	1	2020	69	Single-arm	Solid tumor (*n* = 67), NHL (*n* = 2)	57 (21–80)
ODM-207 (BRDT/BRD2/BRD3/BRD4)	Ameratunga et al. (2020)	NCT03035591	1	2020	35	Single-arm	CRPC (*n* = 12), melanoma (*n* = 5), NMC (*n* = 4), ER^+^BC (*n* = 4), other (*n* = 10)	60 (17–78)

Note: R/R, relapsed/refractory; AML, acute myeloid leukemia; ALL, acute lymphoblastic leukemia; MDS, myelodysplastic syndrome; NHL, non-hodgkin’s lymphoma; NSCLC: non-small cell lung cancer; CRPC, castration-resistant prostate cancer; RA, refractory anemia; CRC, colorectal cancer; NUT, Nuclear protein of the testis; NMC, NUT midline carcinoma; ER^+^BC, estrogen receptor positive breast cancer.

### Quality Assessment

The quality of all the selected studies was evaluated using the MINORS. Considering that all the chosen articles in this study were single-arm and not random, the research quality was scored using the MINORS for non-randomized studies. The result showed all of the studies got satisfactory scores (Supplementary Table S1), except a study of OTX-015 with a score of 12.

### Safety

**FIGURE 2:**
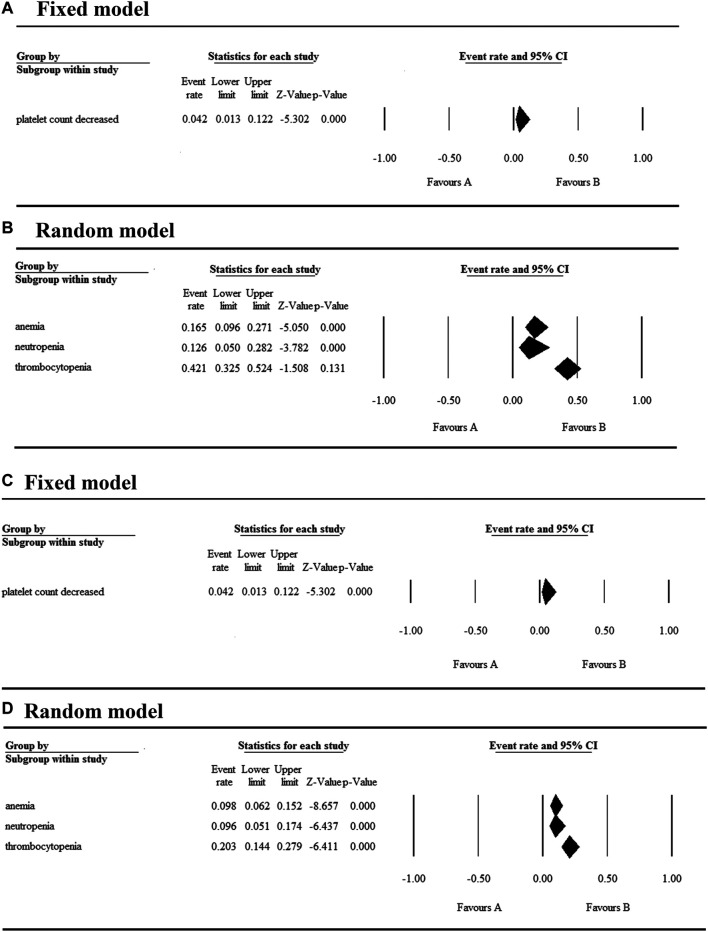
Result of all grade and Grade 3 or 4 hematological AEs in monotherapy with BET inhibitors.

**FIGURE 3:**
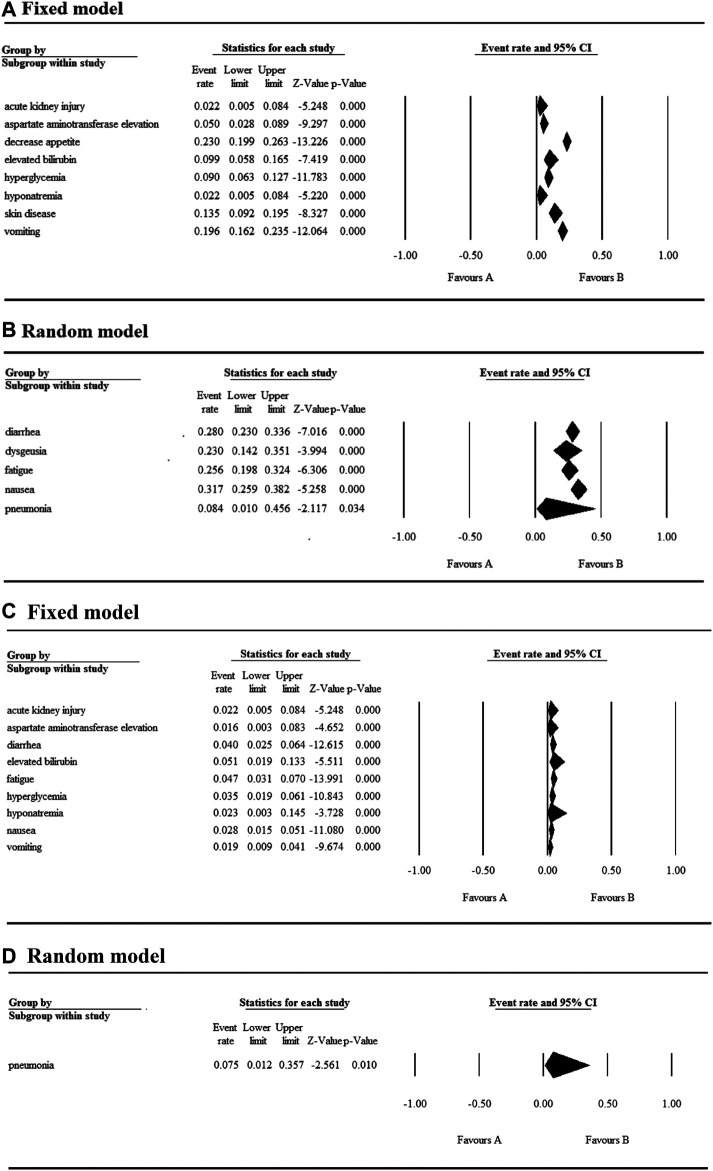
Result of all grades and Grade 3 or 4 non-hematological AEs in monotherapy with BET inhibitors.

**FIGURE 4:**
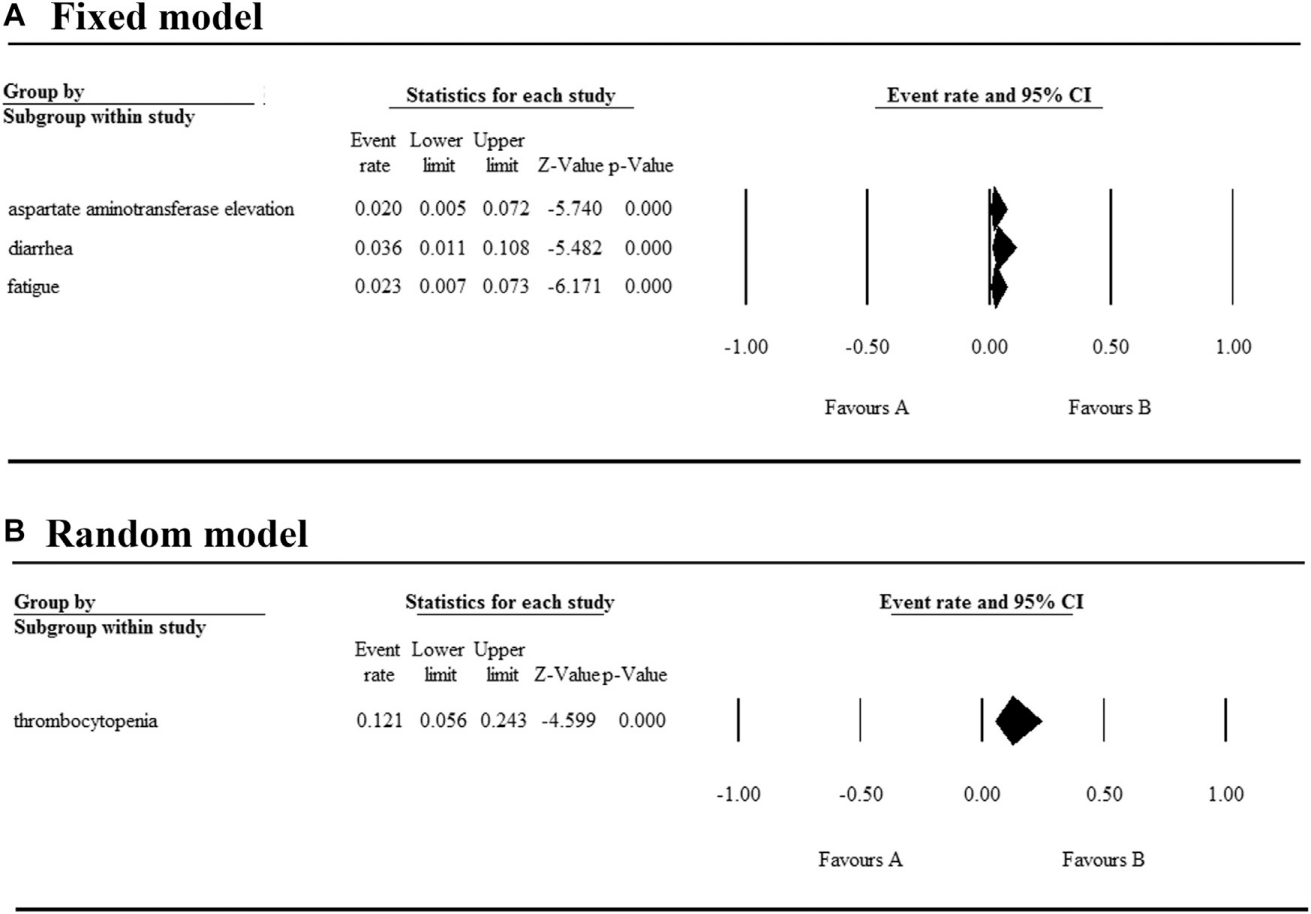
Result of dose-limiting toxicity in monotherapy with BET inhibitors.

The 17 selected articles were single-arm studies and reported adverse events (AEs), their AE rates were calculated and shown in Figures 2–4. The clinical trials of BET inhibitors were performed in patients with hematological malignancies (AML, lymphoma, myeloma, myelofibrosis) and solid tumors (prostate cancer, lung carcinoma, melanoma, breast cancer, sarcoma, NUT midline carcinoma). The top three all grade and Grade 3 or 4 hematological AEs caused by twelve BET inhibitors were thrombocytopenia (42.1%, 20.3%), anemia (16.5%, 9.8%), and neutropenia (12.6%, 9.6%) (Figure 2). Also, the top three all grade non-hematological AEs of the monotherapy of twelve BET inhibitors were nausea (31.7%), diarrhea (28%), and fatigue (25.6%) (Figures 3A,B). However, the top three Grade 3 or 4 non-hematological AEs in the monotherapy of twelve BET inhibitors were pneumonia (7.5%), elevated bilirubin (5.1%), and fatigue (4.7%) (Figures 3C,D). The dose-limited toxicity (DLT) in the monotherapy of these BET inhibitors was mainly thrombocytopenia (12.1%) (Figure 4).

**FIGURE 5:**
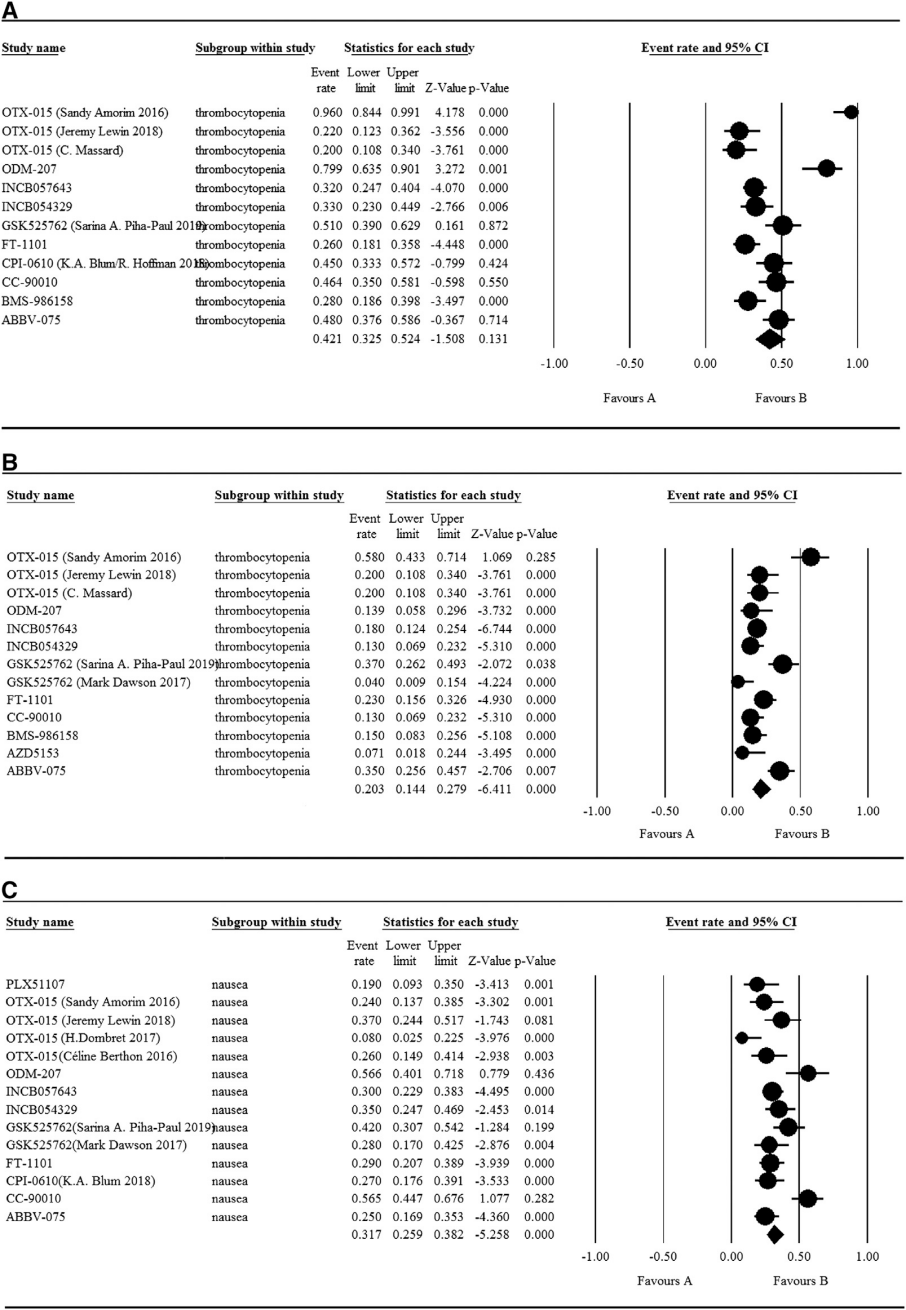
Results of thrombocytopenia and nausea in monotherapy with BET inhibitors.

According to the assessment result of AEs, thrombocytopenia, nausea, diarrhea, and fatigue were the top four AEs of all grades, and thrombocytopenia, anemia, neutropenia, and pneumonia were the most common AEs having Grade ≥3 (Supplementary Table S2). Furthermore, thrombocytopenia ranked first in all grades and Grade ≥3 AEs, as the high occurrence rate correlates to its severity. Eleven articles reported the incidence of thrombocytopenia for twelve BET inhibitors ranging from 20% to 96% (Figure 5A), and the overall and Grade≥3 event rates were 42.1% (95% CI 0.325, 0.524) and 20.3% (95% CI 0.144, 0.279), respectively (Figures 5A,B). However, there were a total of seven patients with hemorrhage, which includes one patient with skin hemorrhage caused by CC-90010, three patients with gastrointestinal hemorrhage caused by ABBV-075, INCB054329, and OTX-015, and three patients with epistaxis caused by OTX-015. Dose intensity was reduced and/or platelet transfusion was performed.

Nausea, reported in thirteen articles, was the second most common AE with an event rate of 31.7% (95% CI 0.259, 0.382) (Figure 5C). Anemia, reported in seven articles, was the second most severe AE with a rate of 9.8% (95% CI 0.062, 0.152). Hyperglycemia, increased bilirubin, and hyponatremia with an overall event rate of <20% were also reported in five, four, and four of the articles, respectively. Additionally, the overall rate of Grade 3 or 4 non-hematological AEs (severe AEs) was below 10% except ODM-207 with 54% and no Grade ≥5 AEs were reported.

As shown in Figure 5, the BET inhibitor with the highest event rates of thrombocytopenia of all grades and Grade ≥3 was OTX-015 reported by Sandy Amorim, while ones with the lowest event rates was OTX-015 reported by C. Massard and GSK525762 reported by M. Dawson. Also, the highest event rate of nausea of all grades was found in the patients treated with ODM-207, whereas the lowest rate was in the patients treated with OTX-015 reported by H. Dombret (Figure 5C).

### Pharmacokinetics

According to the published pharmacokinetic results (Table 2), the steady-state time to reach the maximum plasma concentration (*T*_max_) for these BET inhibitors was between 0.5–6 h, Among them, the BET inhibitor with the shortest *T*_max_ was AZD5153 and that with the longest *T*_max_ was INCB057643 and ODM-207. The terminal elimination half-life (*T*_1/2_) of BET inhibitors were all longer than 10 h except AZD5153 and INCB054329. The longest *T*_1/2_ was observed for BMS-986158, while the shortest was for INCB054329.

**TABLE 2:** Pharmacodynamics of BET inhibitors.

BET inhibitor	Author	*T*_max_	*T*_1/2_
AZD5153	Wang et al. (2019)	0.5–3 h	6 h
ABBV-075	Piha-Paul et al. (2019)	3 h	16.1–19.9 h
BMS-986158	Hilton et al. (2018)	2–4 h	33–82 h
CPI-0610	Blum et al. (2018)	-	16 h
GSK525762	Piha-Paul et al. (2019)	2 h	3–7 h
OTX-015	Lewin et al. (2018)	1–3 h	3–7 h
INCB054329	Falchook et al. (2020)	2–3 h	2.24 ± 2.03 h
INCB057643	Falchook et al. (2020)	2–6 h	11.1 ± 8.27 h
FT-1101	Patel et al. (2019)	2–4 h	30–61 h
CC-90010	Moreno et al. (2020)	1–2 h	51–69 h
ODM-207	Ameratunga et al. (2020)	2–6 h	-

### Efficacy

**FIGURE 6:**
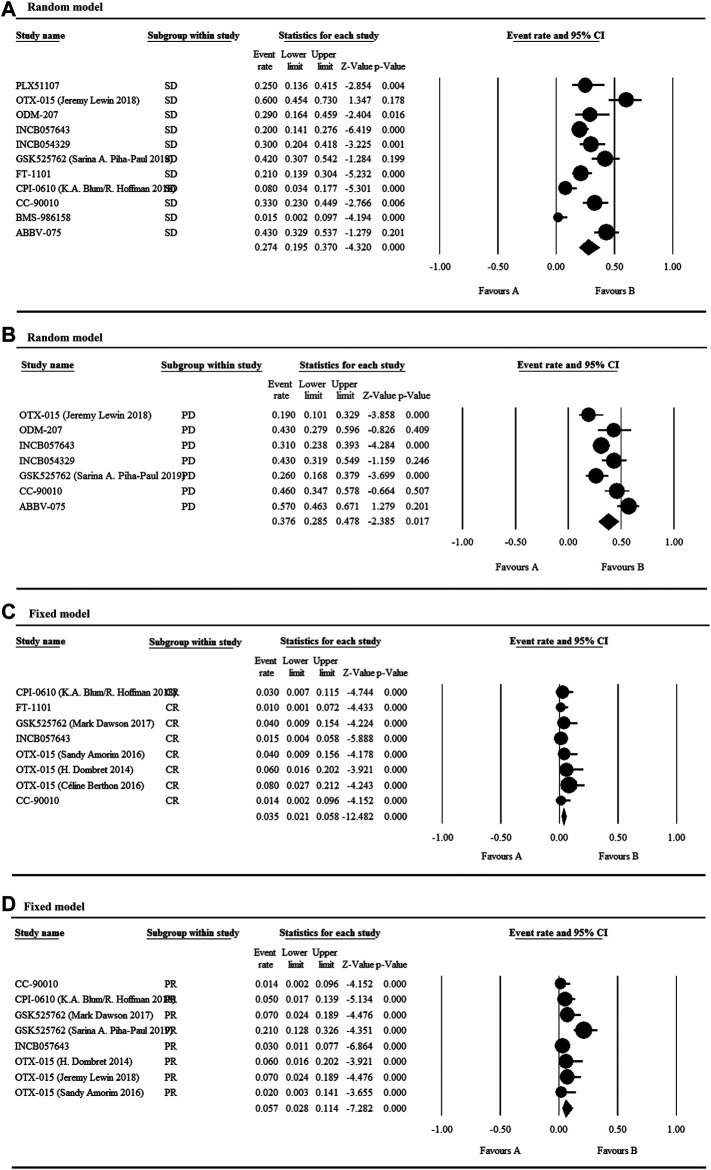
Efficacy of BET inhibitors in the treatment of multiple tumors.

According to current studies, twelve BET inhibitors were applied to treat solid tumor and hematological malignancies. The patients’ overall stable disease (SD) rate with the reported malignancies was 27.4% and the highest SD rate was 60%, which was reached using OTX-015 reported by J. Lewin (Figure 6A). Following that, the second-highest SD was 43%, which was realized by ABBV-075. However, the patients’ overall PD rate was 37.6% and when treating relapsed/refractory solid tumors and prostate cancer using ABBV-075, the patients had the highest PD of 57% (Figure 6B).

The patients’ overall CR rate was 3.5%, whereas the highest CR was 8% after the respective administration of OTX-015 reported by Céline Berthon (Figure 6C). Simultaneously, the patients’ overall PR rate was 5.7% and the highest PR (21%) was observed in the treatment of NUT carcinoma and other solid tumors using GSK525762 (Figure 6D). As shown in Supplementary Table S3 and Supplementary Figure S2, the patients’ overall CR rate negatively correlated with nausea caused by BET inhibitors but exhibited no correlation between the clinical efficacy (SD, PD, CR, or PR) and the most common and grade≥3 adverse effects (thrombocytopenia or nausea).

### Target Genes

**FIGURE 7:**
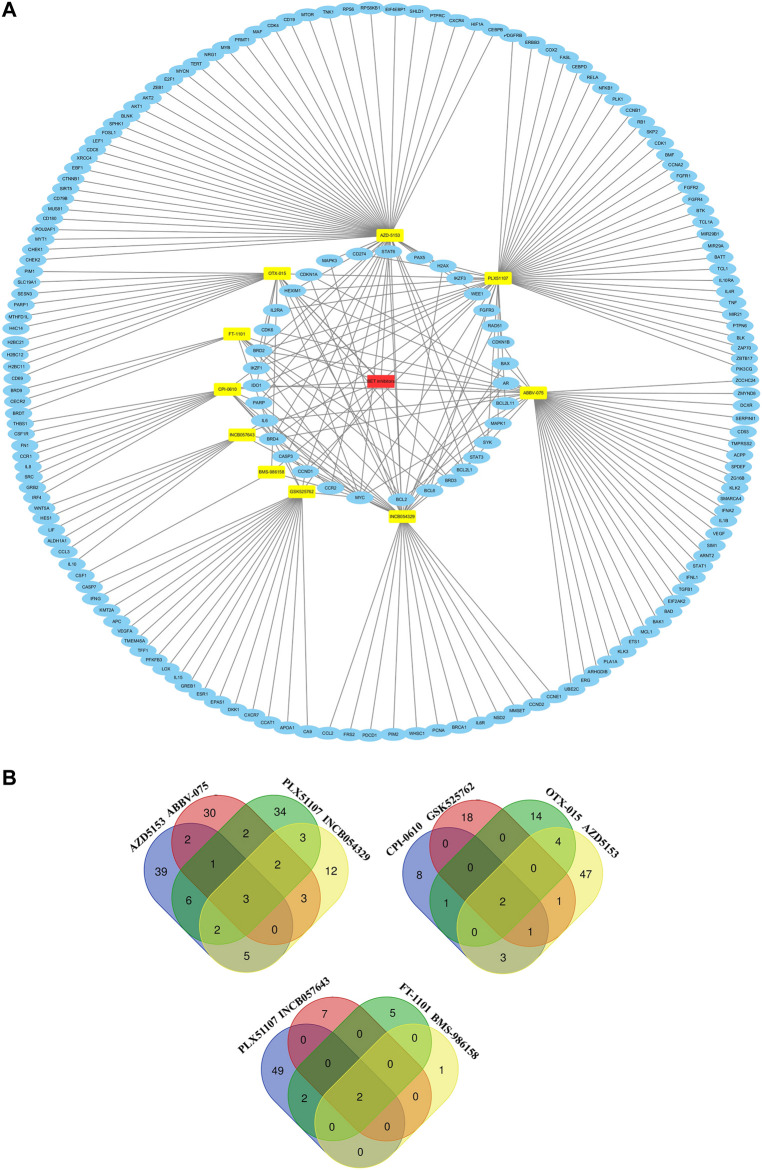
The common and different target genes of 12 BET inhibitors.

**FIGURE 8:**
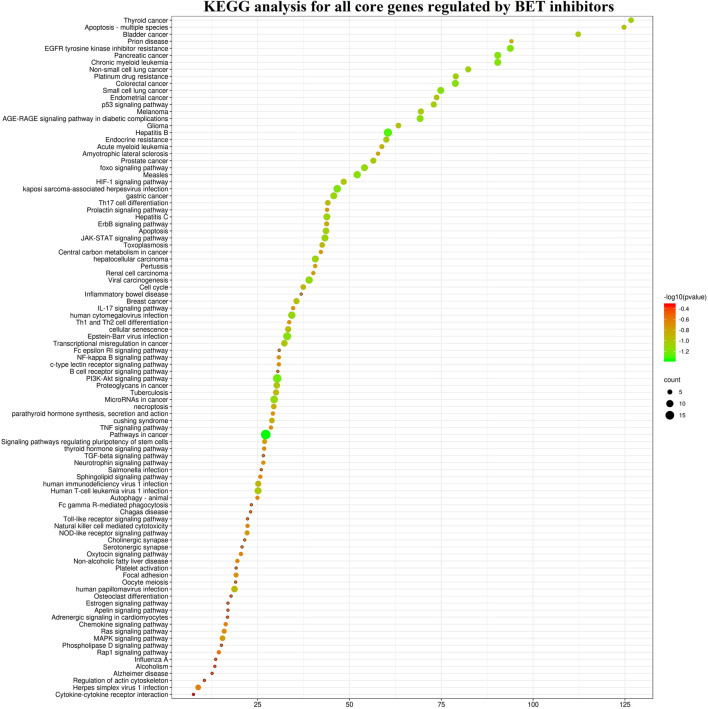
The KEGG pathway enrichment analysis of the core genes for nine BET inhibitors (AZD5153, ABBV-075, BMS-986158, CPI-0610, GSK525762, OTX-015, PLX51107, INCB054329, and INCB057643).

The target genes of these BET inhibitors from published articles were summarized in Figure 7A. Their common target genes as shown in Figure 7 are BRD4 and MYC. Aside from those, the core genes regulated by the nine inhibitors (AZD5153, ABBV-075, BMS-986158, CPI-0610, GSK525762, OTX-015, PLX51107, INCB054329, and INCB057643) are HEXIM1, CASP3, CD274, STAT6, PAX5, H2AX, IKZF1, IKZF3, WEE1, FGFR3, RAD51, CDKN1A, CDKN1B, CDK6, CCND1, CCR2, BAX, AR, BCL2, BCL2L11, BCL2L1, BCL6, MAPK1, MAPK3, SYK, STAT3, BRD2, BRD3, IL2RA, IL6, IDO1, PARP. The KEGG pathway enrichment analysis indicated that these genes participate in pathways in cancer, hepatitis B, PI3K-Akt signaling pathway, kaposi sarcoma-associated herpesvirus infection, EGFR tyrosine kinase inhibitor resistance, chronic myeloid leukemia, pancreatic cancer, JAK-STAT signaling pathway, and many more (Figure 8).

Due to the limited availability of published target genes, the KEGG pathway and GO enrichment analysis could not be performed for FT-1101, BMS-986158, CC-90010, and ODM-207. The KEGG pathway enrichment analysis showed the signal pathways for the other eight BET inhibitors are enriched in cancer and pathogenic microorganism (virus, bacteria, parasite) associated pathways. Among them, the first common signaling pathway was pathways in cancer, which ranked within Top 10 (Supplementary Figures S3–S10). The second and third common ones were hepatitis B and PI3K-Akt signaling pathway, respectively. Additionally, apoptosis was induced by these BET inhibitors AZD5153, ABBV-075, INCB054329, OTX-015, and PLX51107, and the genes including CASP3, FASLG, BAD, BAK1, BAX, BCL2, BCL2L1, MCL1, BCL2L11, PARP1, AKT1, AKT2, MAPK1, MAPK3, NFKB1, RELA, and TNF were involved in this pathway.

The GO analysis showed these BET inhibitors played multiple roles in biological processes and molecular functions (Supplementary Figure S11). The cellular activation, proliferation, apoptosis, differentiation, and activity of various proteins including kinases, which are regulated by these inhibitors, are contributed to the death of tumor cells.

## Discussion

The main biological function of BET proteins is to “read” the N-acetylation of lysine residues of proteins (Filippakopoulos and Knapp, 2014). On one hand, they can bind to the acetylated lysine of histones in the “super-enhancer” (DNA regions enriched with repressive acetylated H3K27 and RNA polymerase II) or promoter regions of the genes (Lovén et al., 2013; Wang et al., 2017); on the other hand, they can interact with the acetylated lysine of transcription factors via bromodomain (Wang et al., 2018). Hence, BET proteins are vital epigenetic regulators of gene transcription and play an important role in the regulation of inflammation, cell cycle, proliferation, and differentiation of cells. Based on their diverse functions, BET proteins also participate in the occurrence and development of multiple tumors (Stathis and Bertoni, 2018).

BRD4 is the most widely investigated BET protein. Since it was first identified as a regulator of gene transcription in 1988, several functions have been elucidated. BRD4 can recruit the PTEFb complex, JMJD6 (a histone arginine demethylase), NSD3 (a histone methyl transferase), P300 (acetyl transferase), and CHD4 (the catalytic subunit of the NuRD chromatin remodeler) (Taniguchi, 2016). BRD4 itself is an atypical kinase that phosphorylates the Ser2 residue of CTD of RNA polymerase II (Devaiah et al., 2012). Besides histones, BRD4 can also interact with acetylated residues in transcription factors (Wu and Chiang, 2007; Alpatov et al., 2014). The most comprehensively characterized transcription factor is nuclear factor-κB (NF-κB), which is regulated by BRD4 via binding to the acetylated lysine-310 residue of its RelA subunit in cancer cells (Huang et al., 2009). The binding of RelA with BDR4 blocks its ubiquitination and inhibits its subsequent proteasome-mediated degradation, leading to the excessive activation of NF-κB and aberrant proliferation of cancer cells (Wu et al., 2013; Zou et al., 2014).

The BET inhibitors have attracted profound interest due to their therapeutic potential and their efficacy was proved in the oncology and non-oncology indications. Until now, BRD4 ranks first in the target of BET inhibitors in the published studies. In this study, BET inhibitors that inhibit the binding of BRD4 to histones are selected. Their safety, efficacy, and pharmacodynamics are systematically analyzed based on registered clinical trials.

With regards to their safety, we analyzed the AEs of patients with hematologic malignancies and solid tumors treated with the monotherapy of BET inhibitors. The most common hematological AEs caused by these inhibitors were thrombocytopenia, anemia, and neutropenia, and they were also the most severe AEs. Thrombocytopenia was observed in approximately 2/5 of the patients and half of the affected patients experienced severe thrombocytopenia. According to the KEGG enrichment analysis result based on published data, we concluded that apoptosis induced by these BET inhibitors may be the main mechanism for thrombocytopenia. Moreover, the most common non-hematological AEs in descending order were nausea, diarrhea, fatigue, dysgeusia, and decreased appetite, while the most severe was pneumonia. The highest event rate of dose-limiting toxicity was thrombocytopenia, whose event rate was less than 15%. Notably, hematological toxicities were the most frequent and severe AE. Therefore, in the treatment of tumors using BET inhibitors, the platelets transfusion or/and dose modification strategy should be performed in urgent situations to prevent the occurrence of fatal complications such as cerebral and gastrointestinal hemorrhage.

Within these BET inhibitors, the event rate of thrombocytopenia caused by OTX-015 were strikingly different among selected articles. Amorim et al. reported thrombocytopenia was the most common and severe AE with an event rate of 96 and 58% (Amorim et al., 2016), respectively, while Lewin et al. found the event rate of thrombocytopenia to be about 22% (Lewin et al., 2018). This difference may be correlated with the type of tumor, as patients with hematological malignancies and solid tumor were treated with OTX-015 in both studies, respectively (Amorim et al., 2016; Lewin et al., 2018). OTX-015 still caused high event rate of nausea with 8%–37%. Therefore, more attention should be paid to the adverse effects when OTX-015 is used in the treatment of tumors, especially hematological tumors. Structural modification was recommended to decrease the adverse effect of OTX-015.

Based on the current data, the patients’ overall CR and PR after monotherapy with BET inhibitors was 3.5% and 5.7%, respectively, demonstrating limited efficacy as a whole. The patient’s highest CR reached 8% when OTX-015 was used to treat acute leukemia (Berthon et al., 2016), indicating the use of BET inhibitors might be more appropriate when applied in the treatment of hematological tumors than solid tumors.

The *T*_max_ of these BET inhibitors was 0.5–6 h, suggesting rapid oral absorption. Furthermore, a high-fat meal had a small delayed effect on *T*_max_, thus BET inhibitors could be administered without regard to meals (Falchook et al., 2020). Additionally, the decreased expression of MYC, an indicator of BET inhibition, was associated with the plasma concentration of BET inhibitors (Falchook et al., 2020). As shown in Table 2, the *T*_1/2_ of BET inhibitors varied significantly. The longest and shortest were 33–82 h of BMS-986158 and 2.24 ± 2.03 h of INCB054329, respectively. However, the relatively short *T*_1/2_ might lead to no clinical response.

According to the current published data, the common target genes of these BET inhibitors were BRD4 and MYC, together they played a vital role in the occurrence and development of tumors. Ninety-seven signaling pathways including cancer, infectious diseases, apoptosis, cell cycle and immunology associated pathways could be regulated by these BET inhibitors. However, the anti-tumor effect induced by BET inhibitors alone might be not strong enough to overcome the tumors.

In all, we have evaluated twelve BET inhibitors in this study. Despite having divergent pharmacodynamics profiles and incomplete clinical response data, all of them showed similar safety outcomes. Their overlapping toxicities were mainly thrombocytopenia and diarrhea, which were simultaneously the most common and severe AEs and dose-limiting toxicity. Additionally, BMS-986158 and FT-1101 exhibited a more favorable pharmacodynamics profile with a longer half-life of ≥30 h. By contrast, INCB054329 had an unfavorable clinical pharmacodynamics profile with the shortest half-life. On the base of the current data, the efficacy of BET inhibitors in the treatment of tumors remains limited, and further efforts are required to explore the optimal combinations to maximize the efficacy.

## Limitations

Here, there are some inevitable limitations to influence the results of our systematic analysis. First, the involved studies were not designed according to the principle of randomized controlled trials. Secondly, the BET inhibitors involved here are in phase I/II of the clinical trials now, or have finished recently, and the data was not published in its entirety, thus the data on efficacy, pharmacokinetics, and pharmacodynamics were analyzed partly in this study. Lastly, these clinical studies enrolled the patients with different types and stages of tumors, which were administered the different drugs, doses, and times, making a retrospective comparison of antitumor activity or clinical response among these drugs not possible.

## Data Availability Statement

The original contributions presented in the study are included in the article/Supplementary Material, further inquiries can be directed to the corresponding authors.

## Author Contributions

YS, ZW, XL, and YS collected data; YS, JH, and YS analyzed and interpreted data; YS and ZH wrote this manuscript; and all authors critically reviewed the manuscript for important intellectual content and approved the final draft.

## Funding

This work was supported by the grants from the National Natural Science Foundation of China (81570157), the Project of Shandong Provincial Natural Science Foundation of China (ZR2020MH379, ZR202011020064) and the Project of Shandong Province Medical and Health Science and Technology Program of China (2018WS077). ZH was sponsored by the Alexander Von Humboldt Foundation, Germany.

## Conflict of Interest

The authors declare that the research was conducted in the absence of any commercial or financial relationships that could be construed as a potential conflict of interest.
