# Supplementary material for: Safety and Efficacy of Bromodomain and Extra-Terminal Inhibitors for the Treatment of Hematological Malignancies and Solid Tumors: A Systematic Study of Clinical Trials
Source: Front Pharmacol. 2021 Jan 26;11:621093. doi: 10.3389/fphar.2020.621093 (PMC7870522; doi:10.3389/fphar.2020.621093)
Supplement: Supplementary file 1 [file datasheet1.docx]

**Safety and efficacy of BET inhibitors for the treatment of hematological malignancies and solid tumors: a systematic study of clinical trials**

*Yanli Sun^1, 2^, Jie Han^3^, Zhanzhao Wang^4^, Xuening Li^3^, Yanhua Sun^5^*, Zhenbo Hu^1, 6^**

**SUPPLEMENTARY FIGURE 1** Chemical structure of 9 reported BET inhibitors.

**SUPPLEMENTARY FIGURE 2 |** Correlation analysis between CR and nausea.

**SUPPLEMENTARY FIGURE 3 |** KEGG enrichment analysis of the target genes of INCB057643.

**SUPPLEMENTARY FIGURE 4 |** KEGG enrichment analysis of the target genes of AZD-5153.

**SUPPLEMENTARY FIGURE 5 |** KEGG enrichment analysis of the target genes of CPI-0610.

**SUPPLEMENTARY FIGURE 6 |** KEGG enrichment analysis of the target genes of GSK525762.

**SUPPLEMENTARY FIGURE 7 |** KEGG enrichment analysis of the target genes of INCB054329.

**SUPPLEMENTARY FIGURE 8 |** KEGG enrichment analysis of the target genes of OTX-015.

**SUPPLEMENTARY FIGURE 9 |** KEGG enrichment analysis of the target genes of PLX51107.

**SUPPLEMENTARY FIGURE 10 |** KEGG enrichment analysis of the target genes of ABBV-075.

**SUPPLEMENTARY FIGURE 11 |** GO analysis of the target genes of 8 BET inhibitors.

.

**SUPPLEMENTARY TABLE 2 |** The research assessment scores according to MINORS

| **Author**  **Publication time** | **A clearly stated aim** | **Inclusion of consecutive patients** | **Prospective collection of data** | **Endpoints appropriate to the aim of the study** | **Unbiased assessment of the study endpoint** | **Follow-up period appropriate to the aim of the study** | **Loss to follow up less than 5%** | **Prospective calculation of the study size** | **Total** |
| --- | --- | --- | --- | --- | --- | --- | --- | --- | --- |
| Judy Sing-Zan Wang, 2019 | ★★ | ★★ | ★★ | ★★ | ★★ | ★ | ★★ | ★★ | 15 |
| Sarina A. Piha-Paul, 2019^17^ | ★★ | ★★ | ★★ | ★★ | ★ | ★ | ★★ | ★★ | 14 |
| J. Hilton, 2018 | ★★ | ★★ | ★★ | ★★ | ★★ | ★★ | ★★ | ★★ | 16 |
| K.A. Blum, 2018 | ★★ | ★★ | ★★ | ★★ | ★★ | ★ | ★★ | ★★ | 15 |
| Marina Kremyanskaya, 2019 | ★★ | ★★ | ★★ | ★★ | ★★ | ★ | ★ | ★ | 13 |
| Sarina A. Piha-Paul, 2019^21^ | ★★ | ★★ | ★★ | ★ | ★ | ★ | ★★ | ★★ | 13 |
| Mark Dawson, 2017 | ★★ | ★★ | ★★ | ★★ | ★★ | ★ | ★★ | ★★ | 15 |
| H.Dombret, 2017 | ★★ | ★★ | ★★ | ★ | ★★ | ★ | ★ | ★★ | 13 |
| CélineBerthon, 2016 | ★★ | ★★ | ★★ | ★★ | ★★ | ★★ | ★★ | ★★ | 16 |
| Sandy Amorim, 2016 | ★★ | ★★ | ★★ | ★★ | ★★ | ★★ | ★★ | ★★ | 16 |
| Jeremy Lewin, 2018 | ★★ | ★★ | ★★ | ★★ | ★★ | ★★ | ★★ | ★★ | 16 |
| C.Massard, 2016 | ★★ | ★★ | ★★ | ★ | ★ | ★ | ★ | ★★ | 12 |
| Amita Patnaik, 2018 | ★★ | ★★ | ★★ | ★★ | ★★ | ★★ | ★★ | ★★ | 16 |
| Gerald Falchook, 2020 | ★★ | ★★ | ★★ | ★★ | ★★ | ★★ | ★★ | ★★ | 14 |
| Manish R. Patel, 2019 | ★★ | ★★ | ★★ | ★ | ★ | ★ | ★★ | ★★ | 13 |
| V. Moreno, 2020 | ★★ | ★★ | ★★ | ★★ | ★★ | ★★ | ★★ | ★★ | 16 |
| Malaka Ameratunga, 2020 | ★★ | ★★ | ★★ | ★★ | ★★ | ★★ | ★★ | ★★ | 16 |

**SUPPLEMENTARY TABLE 2 |** The top four most common AEs of all grades and Grade ≥ 3 in the monotherapy of 12 BET inhibitors

| **AEs** |  | **Any grade** |  |  | **AEs** | **Grade≥3** | | | |
| --- | --- | --- | --- | --- | --- | --- | --- | --- | --- |
|  | **Event rate**  **(95%CI)** | **Z-value** | ***P*-value** | **Statistical**  **method** |  | **Event rate (95% CI)** | **Z-value** | ***P*-value** | **Statistical**  **method** |
| thrombocytopenia | 0.421  (0.325-0.524) | -1.508 | 0.131 | Random | thrombocytopenia | 0.203  (0.144-0.279) | -6.411 | 0.000 | Random |
| nausea | 0.317  (0.259-0.382) | -5.258 | 0.000 | Random | anemia | 0.098  (0.062-0.152) | -8.657 | 0.000 | Random |
| diarrhea | 0.280  (0.230-0.336) | -7.016 | 0.000 | Random | neutropenia | 0.096  (0.051-0.174) | -6.437 | 0.000 | Random |
| fatigue | 0.256  (0.198-0.324) | -6.306 | 0.000 | Random | pneumonia | 0.075  (0.012-0.357) | -2.561 | 0.000 | Random |

**SUPPLEMENTARY TABLE 3** **|** Correlation analysis of clinical efficacy and the most common or grade≥3 adverse effects of BET inhibitors

|  | Thrombocytopenia of all grades | Thrombocytopenia of grade≥3 | Nausea of all grades |
| --- | --- | --- | --- |
| SD | r=0.3091, *P*=0.356 | r=0.2683, *P*=0.4483 | r=0.3333, *P*=0.3487 |
| PD | r=0.3964, *P*=0.3956 | r=-0.3545, *P*=0.4444 | r=-0.1442, *P*=0.7825 |
| CR | r=0.7, *P*=0.2333 | r=-0.1026, *P*=0.95 | r=-0.8024, *P*=0.0218 |
| PR | r=-0.3571, *P*=0.4444 | r=0.2455, *P*=0.5948 | r=0.07186, *P*=0.882 |
