# Supplementary figures and images for: Safety and Efficacy of Bromodomain and Extra-Terminal Inhibitors for the Treatment of Hematological Malignancies and Solid Tumors: A Systematic Study of Clinical Trials
Source: Front Pharmacol. 2021 Jan 26;11:621093. doi: 10.3389/fphar.2020.621093 (PMC7870522; doi:10.3389/fphar.2020.621093)

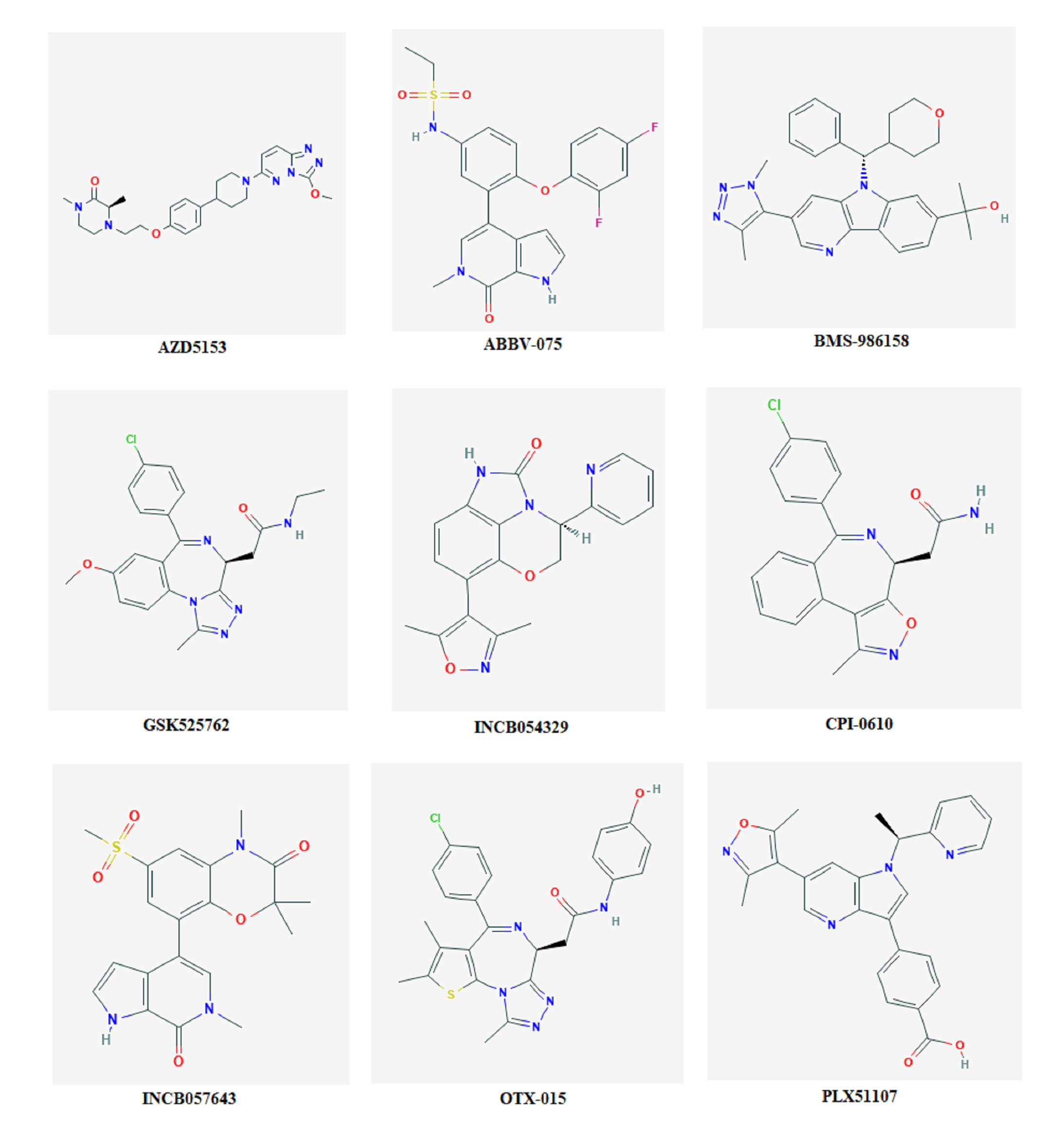

Supplement: Supplementary file 2 [file image1.jpeg]

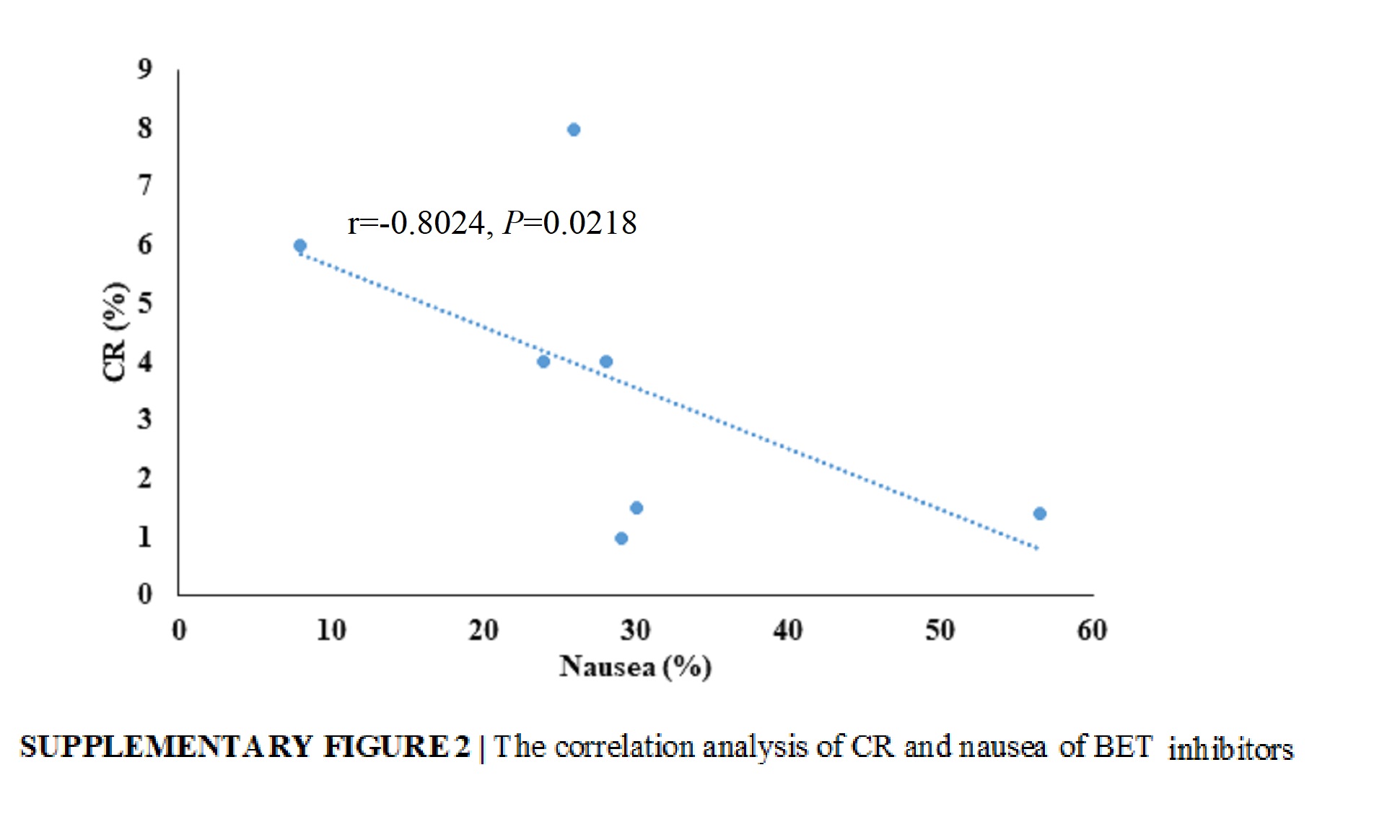

Supplement: Supplementary file 3 [file image2.jpeg]

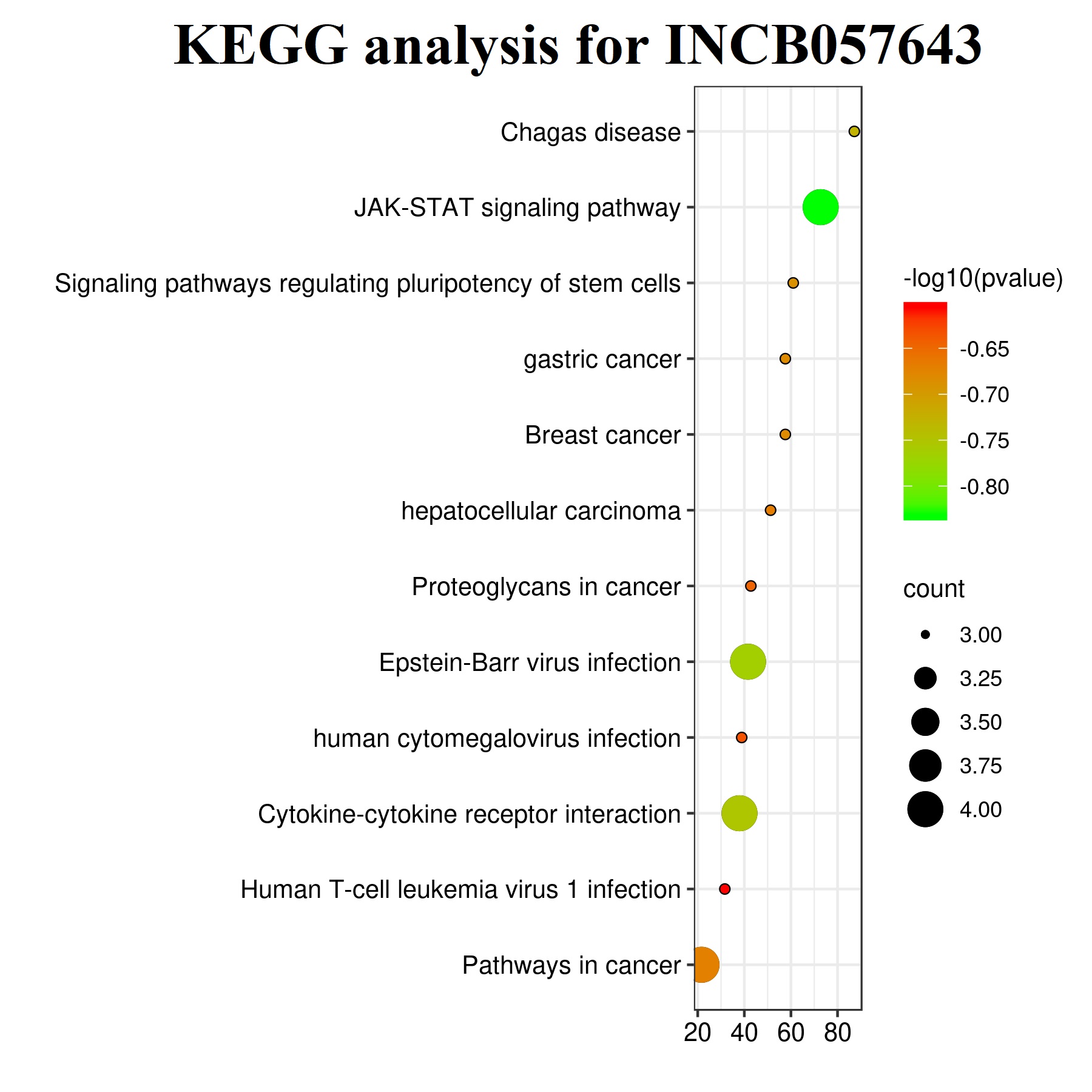

Supplement: Supplementary file 4 [file image3.jpeg]

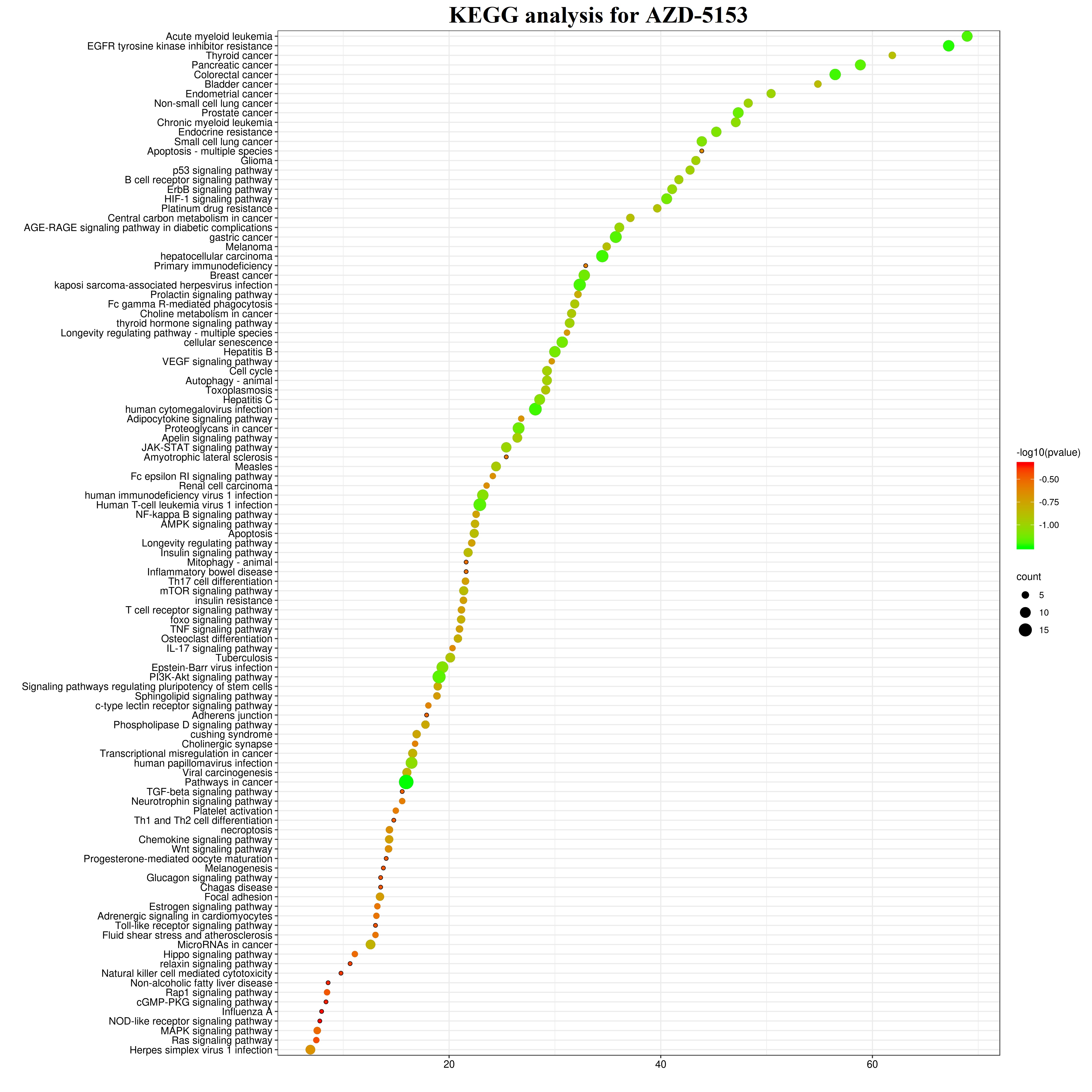

Supplement: Supplementary file 5 [file image4.jpeg]

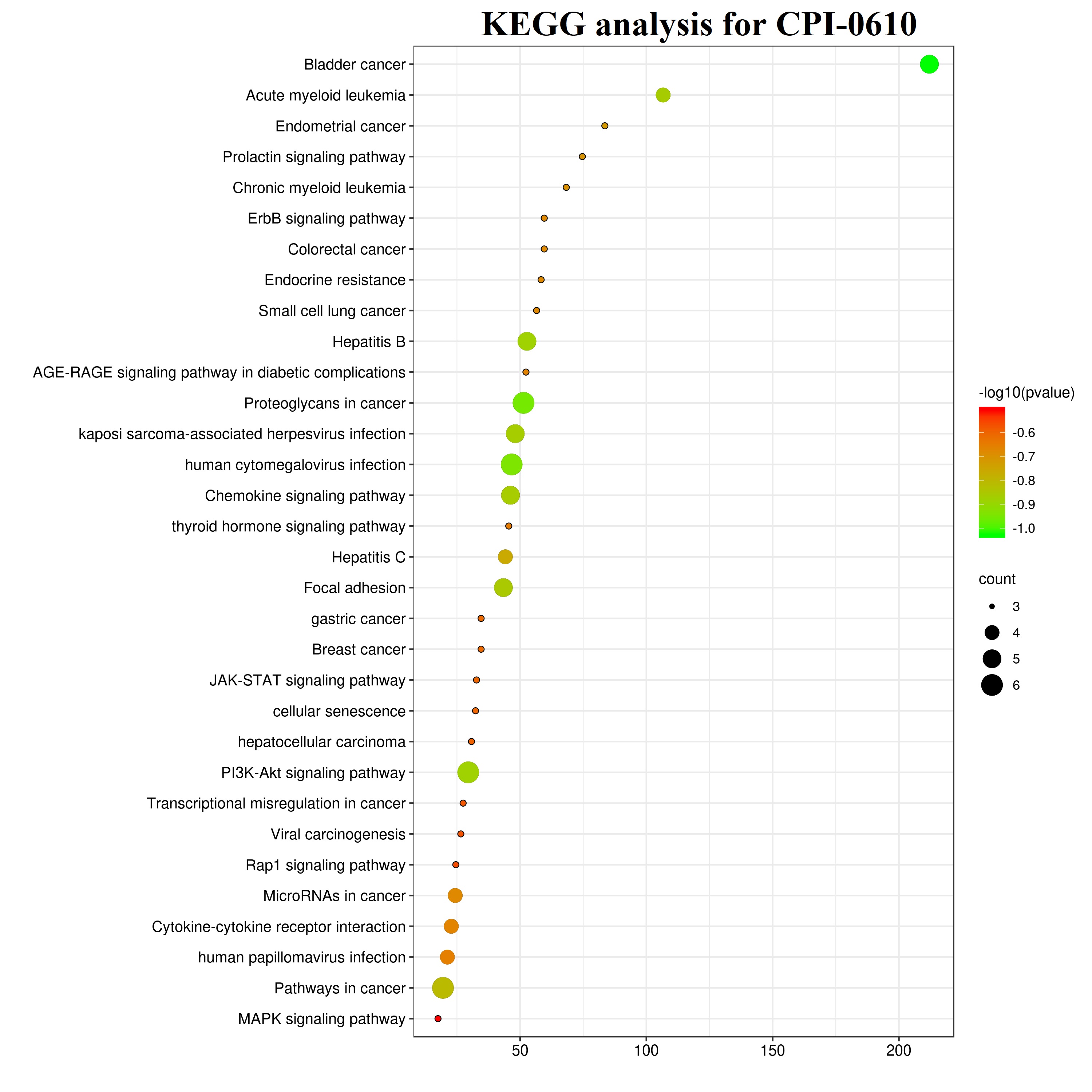

Supplement: Supplementary file 6 [file image5.jpeg]

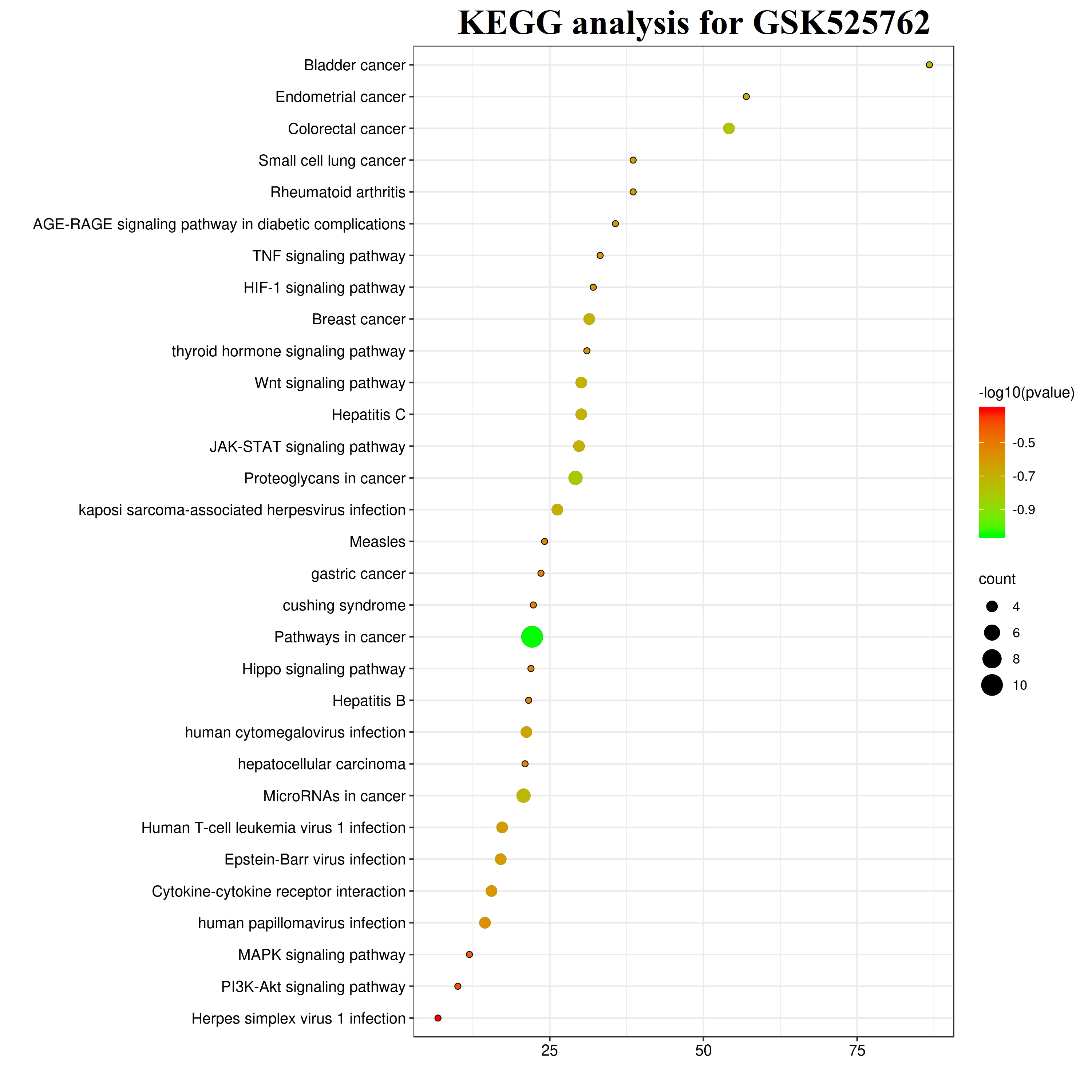

Supplement: Supplementary file 7 [file image6.jpeg]

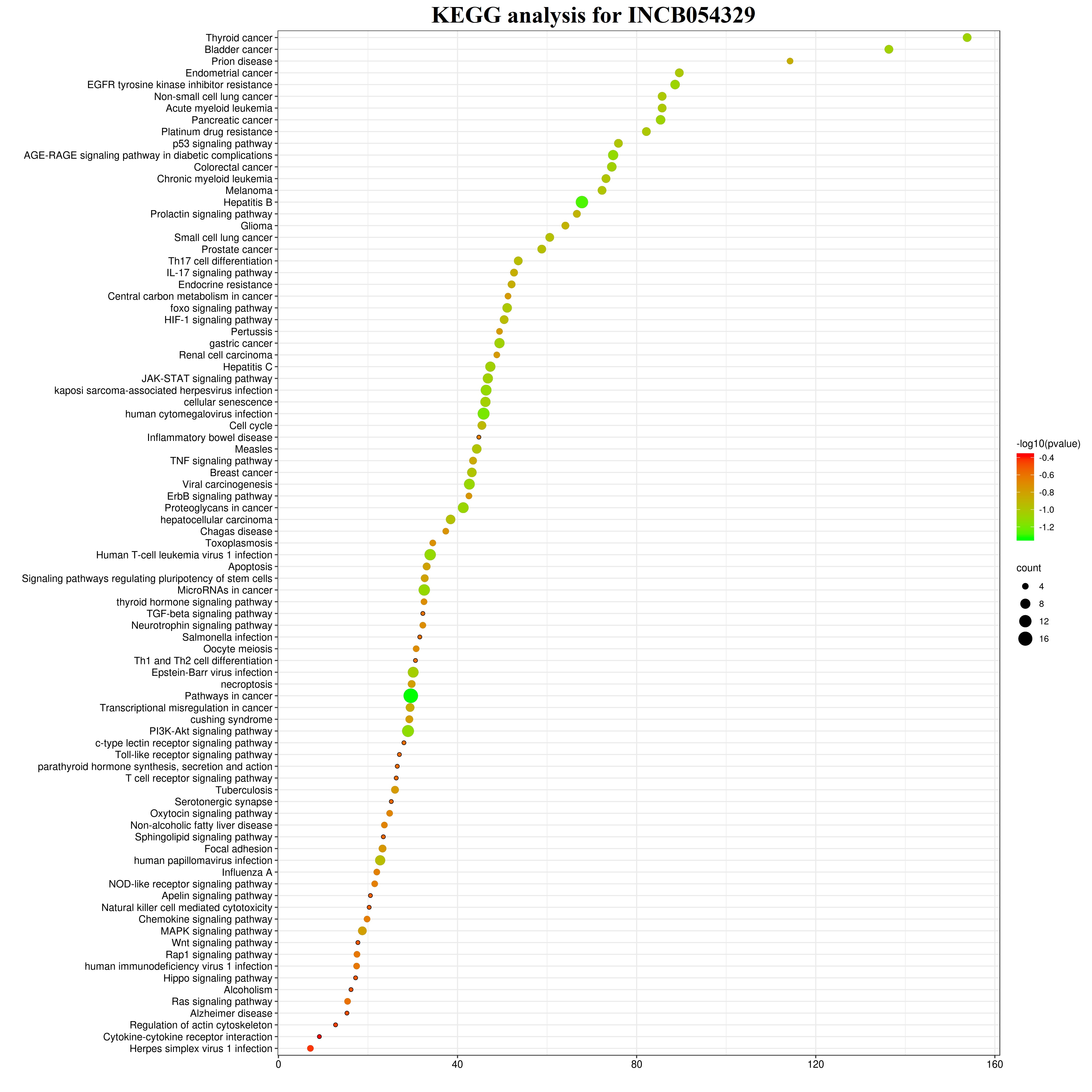

Supplement: Supplementary file 8 [file image7.jpeg]

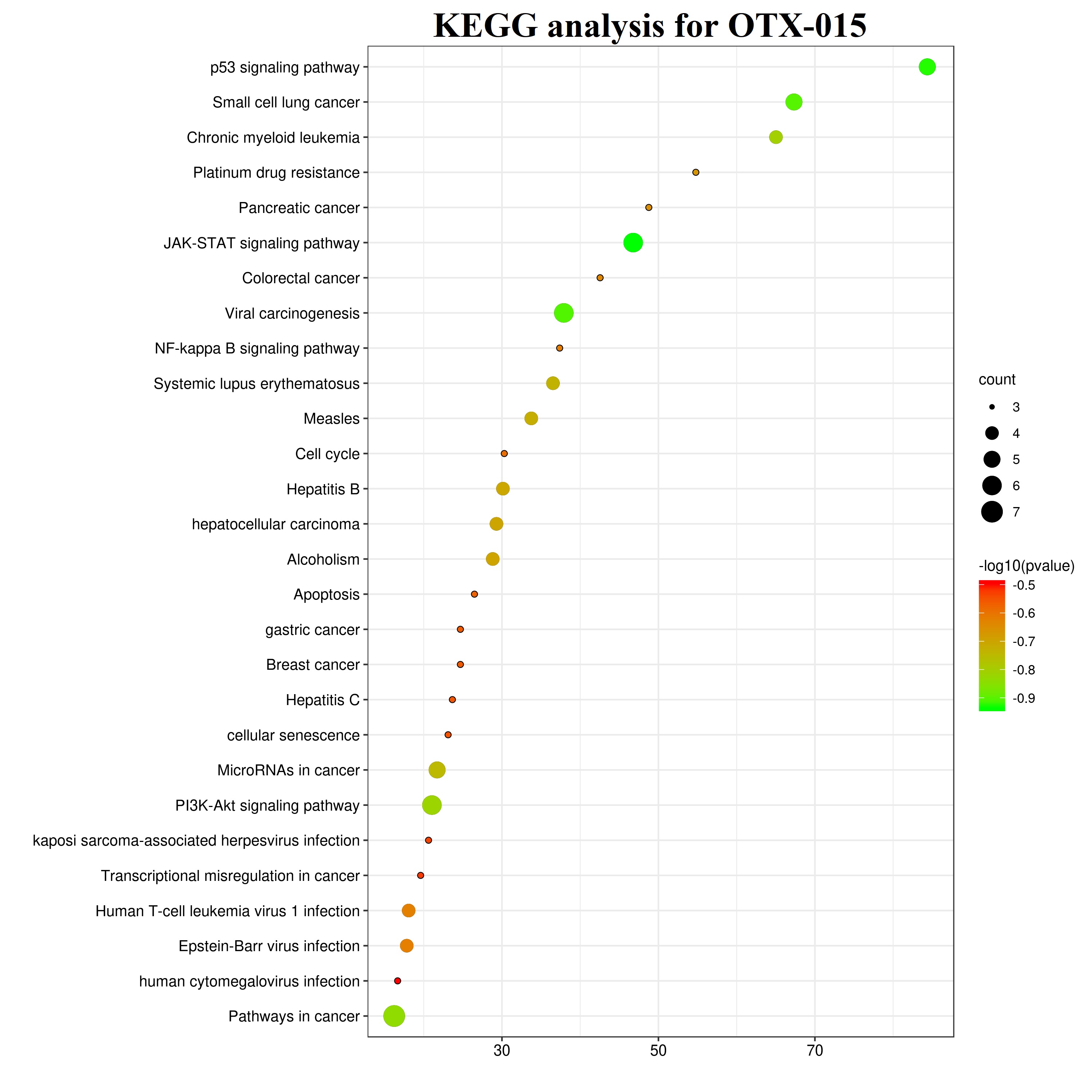

Supplement: Supplementary file 9 [file image8.jpeg]

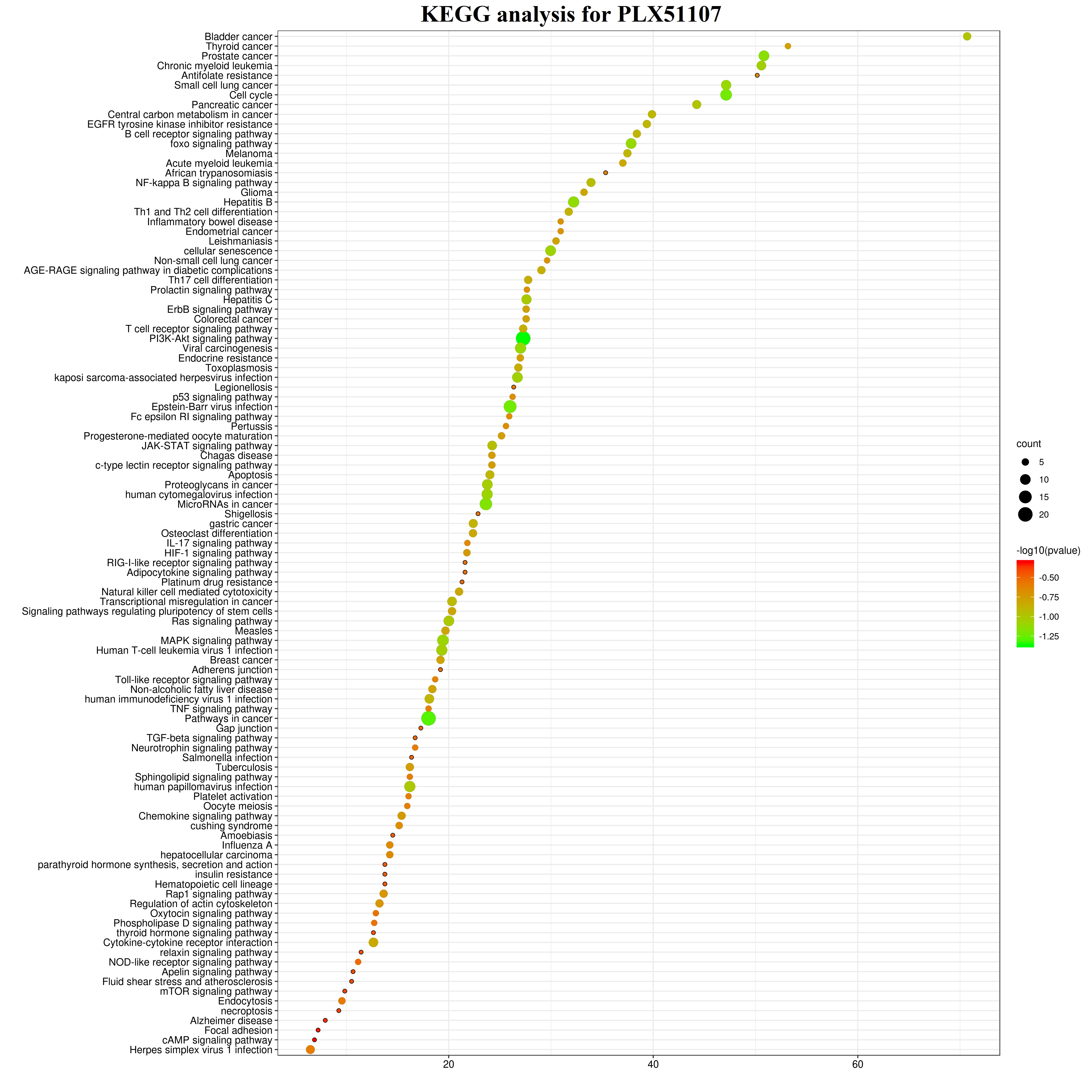

Supplement: Supplementary file 10 [file image9.jpeg]

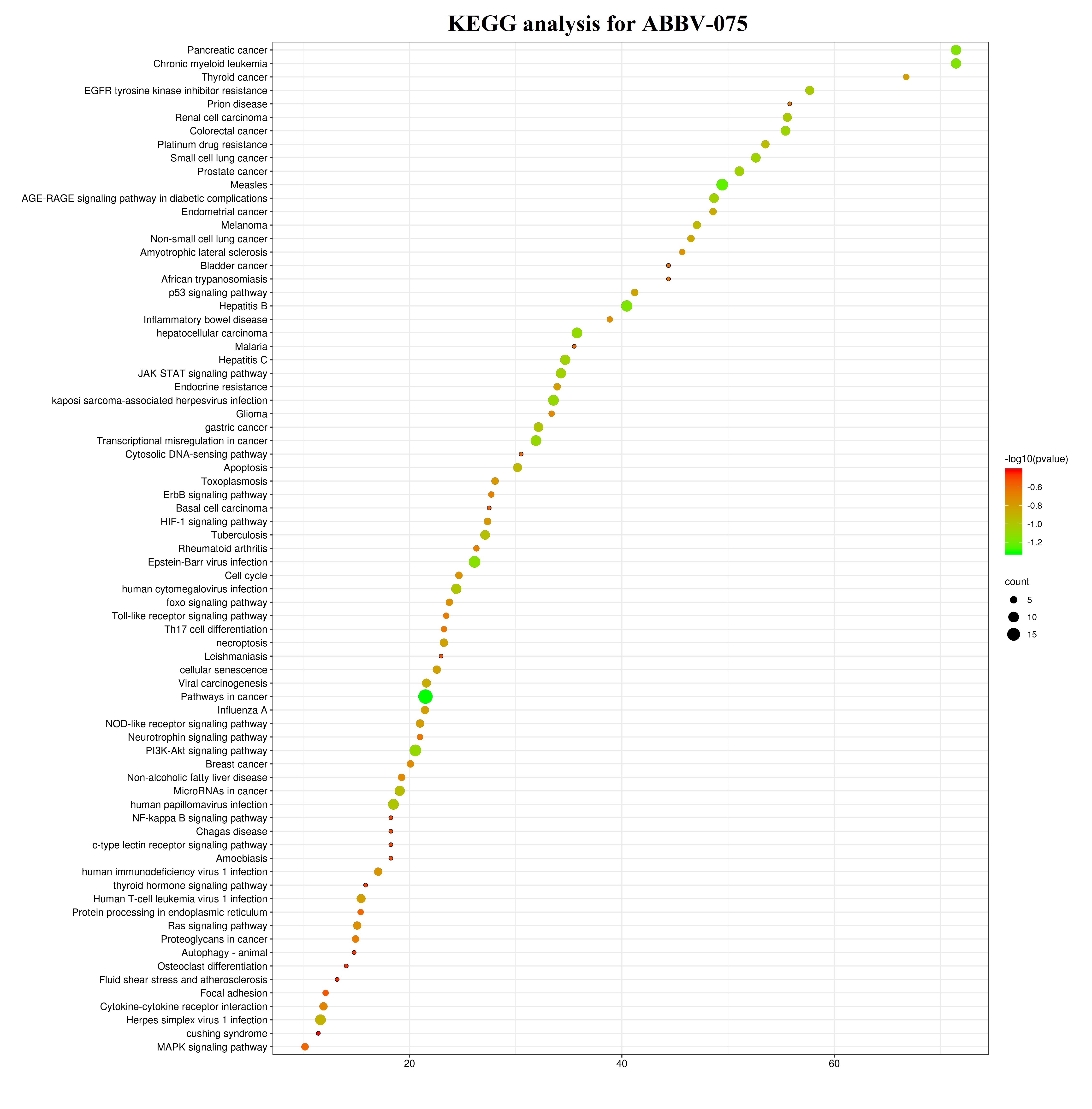

Supplement: Supplementary file 11 [file image10.jpeg]

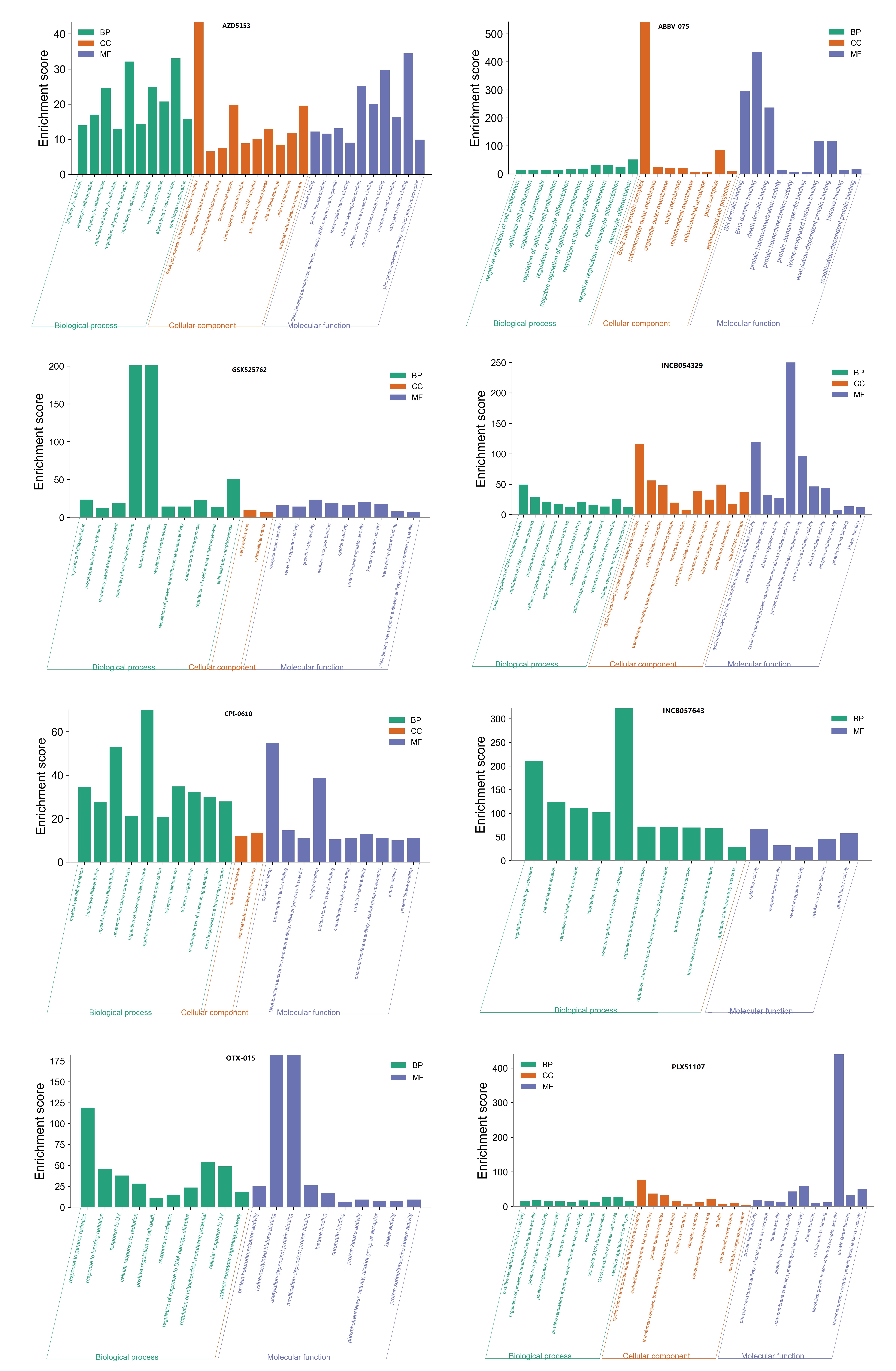

Supplement: Supplementary file 12 [file image11.jpeg]
